# Computer-Assisted Resection and Reconstruction of Pelvic Tumor Sarcoma

**DOI:** 10.1155/2010/125162

**Published:** 2010-11-28

**Authors:** Pierre-Louis Docquier, Laurent Paul, Olivier Cartiaux, Christian Delloye, Xavier Banse

**Affiliations:** ^1^CARS Research Center, (Computer and Robotic-Assisted Surgery), Institute of Experimental and Clinical Research, Catholic University of Louvain, 1348 Louvain, Belgium; ^2^Department of Orthopaedic Surgery, Cliniques Universitaires Saint-Luc, 10, Avenue Hippocrate, 1200 Bruxelles, Belgium; ^3^Center of Research in Mechatronics, Catholic University of Louvain, 1348 Louvain, Belgium

## Abstract

Pelvic sarcoma is associated with a relatively poor prognosis, due to the difficulty in obtaining an adequate surgical margin given the complex pelvic anatomy. Magnetic resonance imaging and computerized tomography allow valuable surgical resection planning, but intraoperative localization remains hazardous. Surgical navigation systems could be of great benefit in surgical oncology, especially in difficult tumor location; however, no commercial surgical oncology software is currently available. A customized navigation software was developed and used to perform a synovial sarcoma resection and allograft reconstruction. The software permitted preoperative planning with defined target planes and intraoperative navigation with a free-hand saw blade. The allograft was cut according to the same planes. Histological examination revealed tumor-free resection margins. Allograft fitting to the pelvis of the patient was excellent and allowed stable osteosynthesis. We believe this to be the first case of combined computer-assisted tumor resection and reconstruction with an allograft.

## 1. Introduction

Patients with malignant neoplasms of the pelvic girdle are at a higher risk for treatment failure than patients with similar tumors located in a limb [1]. The reason for this increased risk is related to the inadequacy of the surgical margin obtained [1, 2] given the complex anatomy in the pelvic area, including the presence of important internal organs and vasculonervous structures [1, 3]. Magnetic resonance imaging (MRI) and computerized tomography (CT) are valuable tools for locating tumors and for surgical resection planning, but it remains difficult to transfer this valuable information to the operating room. 

During surgery in complex anatomical locations (e.g., pelvis or sacrum), the surgeon is often lost [4] and lacks precision. Inadequate resection margins (intralesional or marginal) are frequently obtained, leading to local recurrence [2, 5, 6]. Local recurrence rates for pelvic osteosarcoma of up to 70% (marginal margins) or 92% (intralesional margins) have been reported [5]. Pelvis reconstruction with an allograft is further complicated by the difficulty of cutting the bone allograft to precisely fit the resection gap [2, 7]. This procedure also lacks in precision, leading to discrepancies at the host-allograft junctions and increased risks of osteosynthesis failure and nonunion.

Computer-assisted surgical navigation systems that aid in surgical precision are widely available for procedures such as total hip or knee arthroplasty, anterior cruciate ligament reconstruction, high tibial osteotomy, and pedicle screw placement in spine surgery [8]. Additional computer-assisted applications specific for the pelvic region include those for osteotomies and for the placement of sacroiliac screws or long lag screws in the osteosynthesis of acetabular fractures [9, 10]. Surgical oncology could benefit from navigation technologies for tumor resection and pelvis reconstruction. Despite a lack of commercially available navigation software for musculoskeletal tumor resection, navigations have been performed for pelvic and sacral tumor resections [1, 3, 11, 12]. In these cases, commercially available software programs were adapted to navigate the axes, but no direct plane navigation was performed. In combination with MRI and CT, computer-assisted navigation could facilitate intraoperative localization and improve safety during resection. 

To date, navigation software has never been used for simultaneous resection and allograft reconstruction. We report a case of synovial sarcoma involving the acetabular area of the pelvis (zone II and III of Enneking) [13] in which tumor delineation was performed using MRI and MRI-to-CT coregistration, as well as combined navigation of the tumor resection and graft cutting.

## 2. Patient and Method

### 2.1. Patient

A thirty-two-year-old man was referred to our department for a longer than one-year history of hip pain. Neurological deficits and systemic symptoms, such as fever or weight loss, were absent. A plain radiograph of the pelvis showed an osteolytic lesion of the ischium. Computed tomography and MRI revealed a tumor involving the acetabular area, ischium, and pubis. Further evaluation, including chest CT and PET-CT (fluorodeoxyglucose-18) showed no distant metastases. An incisional biopsy was performed and histological evaluation of the biopsy tissue was consistent with the diagnosis of grade III synovial sarcoma.

### 2.2. Preoperative Planning for Tumor Resection

**Figure 1:**
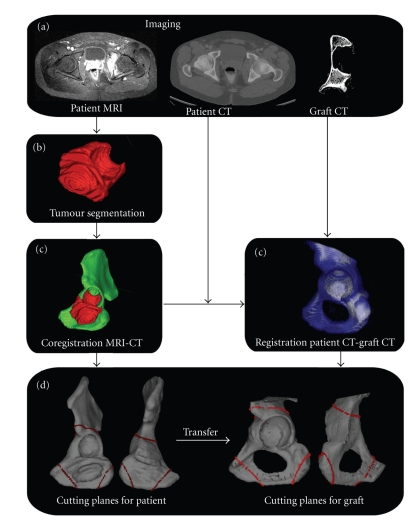
(a) Preoperative imaging workup of the patient consisted of MRI and CT of the pelvis. A CT of the allograft (iliac bone) was also obtained. (b) The tumor is segmented on the MRI slices. (c) The MRI and CT are coregistered. The CT of the patient and of the allograft are registered. (d) The target planes (red lines) are preoperatively defined by locating the planes with respect of a safe margin from the tumor. The planes coordinates are transferred to the allograft.

Preoperative pelvic CT and MRI scans were obtained using a Brilliance 40 CT scanner (Philips, the Netherlands) with a 1-mm spacing between slices and 2-mm slice thickness and a 1.5 T NTScan Intera MRI (Philips, the Netherlands) with a 4-mm spacing between slices, 3-mm slice thickness, repetition time of 550 msec, and echo time of 14 msec. The open-source software ITK-Snap 1.6.0.1 (www.itksnap.org/) was used to manually segment the tumor on each MRI slice where it was visible (Figures 1(a) and 1(b)). Since MRI and CT have different locations, image sizes, and resolutions, the MRI-segmented tumor needed to be exported to CT. Coregistration enabled location of the MRI and segmented tumor volume into the CT coordinate system (Figure 1(c)). 

For pelvis osteotomies, three target planes were planned to ensure resection of the whole tumor volume with a tumor-free margin. The first plane was horizontal above the acetabulum, the second was vertical in the pubis, and the third was coronal in the ischiatic tuberosity (Figure 1(d)). These target planes were positioned in three dimensions (3D) on the CT scan using a haptic device [14]. The planes were initially brought into contact with the tumor and then translated back with a surgeon-defined security margin of at least 5 mm [15].

### 2.3. Preoperative Planning for Allograft Cutting

To select the best-fitting allograft among the available iliac bone allografts from the local bone bank, we performed a CT-based (spiral Elscint Twin CT scanner) registration between the iliac bone of the patient and different allografts [16]. The selection criteria were the best congruency of the two iliac bones and their acetabula. Once the optimal allograft was chosen, the CT-to-CT registration permitted us to transfer the target plane CT coordinates of the patient to those of the allograft (Figure 1(d)). Use of this procedure ensured that the target planes for the patient and allograft were identical.

### 2.4. Navigated Surgery Rehearsal with Prototypes

**Figure 2:**
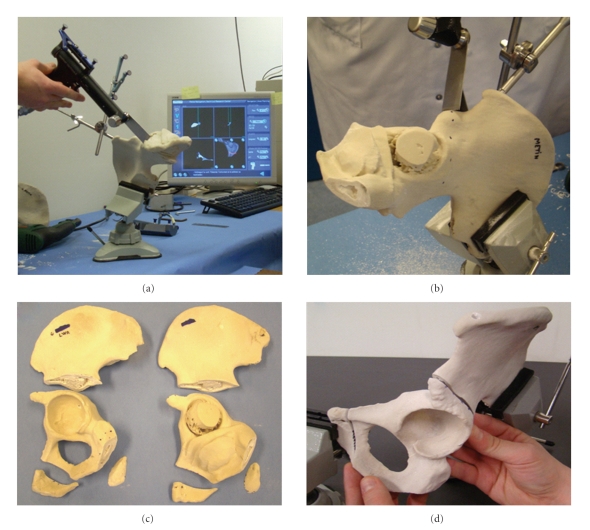
(a) A model of the patient's iliac bone with the tumor was created by rapid prototyping. A preoperative rehearsal of the tumorous resection was realized with the computer-assisted navigation system. (b) Prototype of the patient with the tumor. The osteotomy line was first traced with the navigation pointer to mark up the entry point of the saw. (c) Computer-assisted system allowed us to cut the 2 iliac bone prototypes: the patient (on the right) and the allograft (on the left). The cutting planes are identical for the two procedures. (d) The fitting of the graft model into the patient's iliac bone model is checked.

Models of the iliac bone of the patient and of the selected allograft were created by rapid prototyping [17] (Sirris, Liège, Belgium) (Figure 2(a)) using a 3D plaster printing based on the CT data. Two days before the operation, the surgeon rehearsed tumor resection with target plane navigation (Figure 2). The pelvic CT data of the patient, including the target planes and tumor volume segmentation, were loaded into the navigation system (a customized version of Spineapplication, 1.4, Praxim, LaTronche, France). 

A dynamic reference-base (DRB) was fixed to the iliac crest of the prototype using a rotational stabilizer and was tracked by an NDI Polaris localizer (NDI, Waterloo, Ontario, Canada). Prototype-to-image matching was performed with a surface-based matching algorithm by palpating both the ilium and pubis of the prototype. Error of registration was verified by palpating different points of the pelvis. The saw blade was calibrated in 2D with a developed calibration frame. The three preoperatively defined target planes were marked with the navigation pointer, providing a start line for the saw (Figure 2(b)). The saw blade was then navigated to follow the predefined target plane, and three osteotomies were performed. Graft cutting was rehearsed before the operation with navigation of the same target planes using the system above (Figure 2(c)), and allograft fitting in the iliac bone defect of the patient was verified using the two cut prototypes (Figure 2(d)).

### 2.5. Navigated Surgery

**Figure 3:**
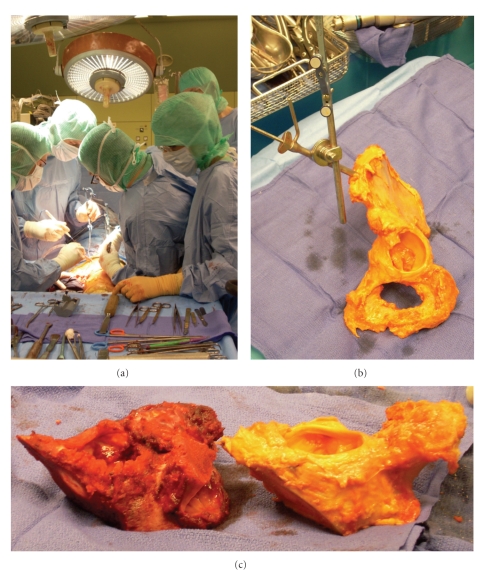
(a) Computer-assisted resection of the tumor. (b) Computer-assisted cutting of the allograft. (c) Tumor resected with safe margins (left) and allograft (right).

The patient was placed in the lateral position, and ilioinguinal and Kocher-Langenbeck approaches were used [18]. After soft-tissue dissection, the DRB was fixed to the iliac crest. Patient-to-image matching was performed by palpating both the ilium and pubis [19]. Match quality was checked by palpating some points with the navigation pointer on the available surface. The three defined target planes were marked and cut with the navigated saw blade (Figure 3(a)). 

**Figure 4:**
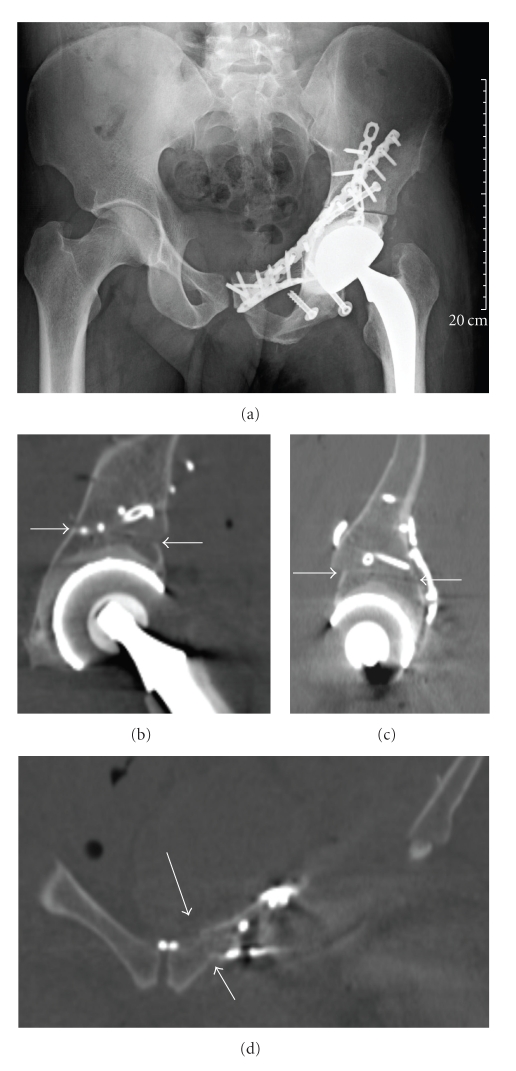
(a) Postoperative radiograph of the pelvis showing the reconstruction. (b) CT with coronal reconstruction showing the host-graft junction (white arrows) in the supraacetabular area. (c) Sagittal reconstruction of the supraacetabular junction (white arrows). (d) Coronal reconstruction of the pubic junction (white arrows).

The DRB was fixed to the iliac crest of the chosen allograft (Figure 3(b)), graft-to-image matching was performed, and match quality was again verified by palpating points at the surface of the pelvis. The error of registration was measured by the root mean square (RMS). RMS was calculated from 520 points used for surface matching and was 0.42 mm. The same three target planes as used for tumor resection were navigated by direct saw navigation. The cut allograft (Figure 3(c)) was placed in the bone defect, and osteosynthesis was performed with three titanium plates (Figure 4(a)). One plate each was placed over the innominate line on the anterior column, over the pubis, and over the posterior column. An additional lag screw was inserted from anterior to posterior in the superior host-graft junction. The ischium was osteotomized (without navigation) with the muscle insertions of the patient and reinserted on the graft with two percutaneous lag screws. Total hip arthroplasty was performed between the graft acetabulum and patient femur.

The total surgery time was 13 h 4 min from the time of skin incision to the end of skin closure. Preoperative preparation took 1 h 22 min, including general anesthesia, thoracic epidural catheter insertion, patient positioning, and draping. The estimated blood loss was 4.1 L. Hemostasis difficulties were encountered close to the ischiopubic ramus due to the proximity of the corpus cavernosum.

### 2.6. Postoperative Period

Histological examination of the removed sarcoma revealed a synovial sarcoma, and showed that all resection margins were tumor-free. The following day, the patient underwent surgery for hemostasis. The patient developed transient femoral nerve paresis, as confirmed by electromyography. The patient walked on crutches with partial weight-bearing for three months, after which full weight-bearing was allowed. A postoperative CT scan revealed perfect plane-to-plane contact between the allograft and host pelvic bone (Figures 4(b), 4(c), and 4(d)).

## 3. Discussion

Dedicated tumor navigation software modules are nowadays available for commercial use. No specific navigation software has been developed to support both tumor resection and reconstruction with navigation of a sawblade. Therefore, we adapted the spine module of a commercially available navigation system (initially developed for pedicle screw application) to enable plane navigation. Although similar adaptations have been previously reported [3, 11], these programs have navigated axes, not planes. 

### 3.1. Preoperative Planning for Tumor Resection

Both CT and MRI are essential for tumor surgery preoperative preparation. A CT reveals precise bony details, while an MRI is superior in delineating the intraosseous and extraosseous tumor extensions, particularly in the soft tissues [20]. For synovial sarcoma, MRI is also the preferred imaging module [21]. In the present study, tumor segmentation was only possible using MRI because the tumor had an extraosseous extension. Accurate tumor delineation was made on each MRI slice where the tumor was visible. Suspicious tissue on MRI was considered pathological and was segmented as tumor. The MRI slice thickness was 3 mm and the interval between slices was 4 mm. A safe margin of more than 5 mm was, therefore, chosen to ensure to overcome the potential error of 3 mm due to MRI slice thickness. Because our navigation system was based on CT, it was necessary to export the tumor volume segmentation by MRI-to-CT coregistration. Image coregistration is effectively used in radiotherapy [22], where the additional information provided by MRI or positron emission tomography can be combined with the CT. Computer-assisted surgery with MRI-to-CT coregistration has been established for the resection of intracranial, craniofacial, and orbital tumors [23, 24], as well as for percutaneous radiofrequency ablation of cancer [25] and for neurosurgery [26]. However, this technology is used rarely in orthopedic oncology.

### 3.2. Preoperative Planning for Allograft Cutting

The CT-based registration method of allograft selection offers several advantages. First, selection of the best-fitting allograft is more efficient than when using the radiograph-based method with templates [27]. Graft-to-patient registration provides a 3D-imaging of the superimposed graft and the pelvis of the patient, which is useful for precisely choosing the graft according to the graft and acetabular sizes and the correspondence of the pubis and sacroiliac joint. Graft-to-patient registration also allows the transfer of the target plane coordinates from the patient to the graft, thus guaranteeing that the cut allograft perfectly fits the bone defect. Graft cutting without a navigation system reportedly produces important gaps at the graft-host junction that could potentially interfere with union and stability [7].

### 3.3. Navigated Surgery Rehearsal with Prototype

Surgery rehearsal with prototypes allowed us to choose the best location of the DRB, thereby increasing rigid body visibility. The target plane feasibility, determined as whether or not the saw blade could reasonably reach the target plane through the planned surgical approach, was also checked. An allograft fitting test within the patient defect showed a small gap due to bone loss related to the saw blade thickness. It was decided to move the osteotomy planes 1.5 mm back in the graft to compensate for this bone loss.

### 3.4. Navigated Surgery

Simultaneous use of ilioinguinal and Kocher-Langenbeck approaches provides improved exposure, thereby facilitating reduction and fixation in acetabular fractures [18, 28], tumor resection, and reconstruction. The optimal DRB location, determination of which increases the match quality [19], was located in the posterior iliac crest to maintain constant visibility by the optical localizer and maximize distance from the working area. A rotational stabilizer was used to increase the marker stability compared with a single screw [29]. Using an ilioinguinal approach, we previously observed a global navigation system accuracy of 1.3 mm [19]. 

In computer-assisted surgeries, there are two methods of surgical planning: preoperative (generally CT image based) and intraoperative (generally fluoroscopy based) [30]. Intraoperative planning, such as using fluoroscopy C-arm technology to navigate a chisel with a cutting block [31] to perform high tibial open-wedge osteotomy, does not require a preoperative CT scan, but it is time consuming. In pelvic surgery, patient installation using a combined approach does not allow for easy intraoperative fluoroscopy. Therefore, we performed preoperative CT-based surgical planning to decrease the operating time and avoid intraoperative fluoroscopy. Since CTs are routinely obtained in surgical oncology, we did not need to obtain additional scans.

Most navigation systems capable of navigating a saw blade only help in cutting block alignment. However, cutting blocks are bulky instruments. Haider et al. developed free-hand cutting with an oscillating bone saw in total knee arthroplasty. When the entire saw was registered as one rigid body, the free-hand technique was found to be 15% faster than using conventional cutting blocks. The implant fit was 400% better with the navigated free-hand bone cutting than that obtained using cutting blocks [32], probably due to error in positioning the cutting block or slight shifts in the cutting block location during cutting.

## 4. Conclusions

**Figure 5:**
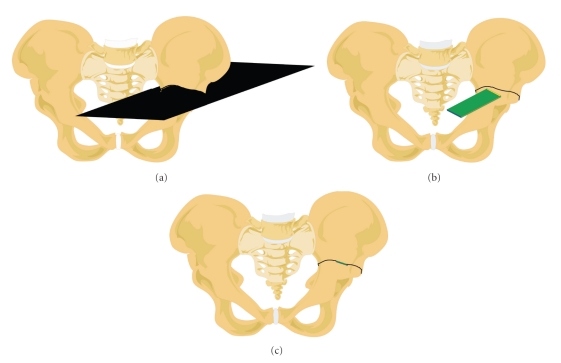
(a) Horizontal target plane horizontal above the acetabulum. (b) Only the intersection of the target plane with the pelvis is represented as a black line at the surface of the pelvis. The saw blade is visualized as a rectangle. The distal edge of the blade is represented by the yellow line and its proximal edge by a blue one. (c) When the saw blade is perfectly orientated into the resection plane, the three lines are perfectly aligned.

We believe this to be the first reported case of combined computer-assisted tumor resection and reconstruction with an allograft. To our knowledge, the patient-to-graft transfer of plane coordinates has never been reported. This technique allowed us to cut the allograft simultaneously (by another surgeon) or before tumor resection, since the planes were defined preoperatively, thereby decreasing the operating time. The accuracy of intraoperative identification by navigation allowed us to obtain a safe margin by avoiding unnecessary resection. This was also the first combined clinical use of a free-hand saw for both tumor resection and reconstruction. The iliac bone, tumor, target planes, and saw blade were directly visualized in 3D on a computer monitor in the operating room (Figure 5). For iliac target planes of >10 cm, a slight error of even 5° in plane orientation leads to a noticeable gap at the host-graft junction. With computer assistance, precise tumor sectioning and allograft cutting led to good contact in the host-graft junctions (Figure 4).

The purpose of limb salvage surgery is to preserve a functioning limb. The surgery requires that the surgeon goes closer to the tumor than they would in amputation, but without increasing patient risk. Osteosarcoma studies have shown an increased risk of local recurrence in patients undergoing limb-salvage surgery, related to the excision margins and the responsiveness of the primary tumor to chemotherapy [33]. Strict respect of safe margins is therefore crucial. Technologies such as those described in the present study may help to improve patient safety and surgical precision in surgical oncology.

##  Ethical Prerequisite

The report of this case has been approved by an ethical committee (registering no. B40320095697).

##  Conflict of Interests

The authors state that there is no conflict of interests.

## References

[1] Hwan SC, Hyun GK, Kim HS, Han I (2008). Computer-assisted sacral tumor resection: a case report. *Journal of Bone and Joint Surgery A*.

[2] Delloye C, Banse X, Brichard B, Docquier PL, Cornu O (2007). Pelvic reconstruction with a structural pelvic allograft after resection of a malignant bone tumor. *Journal of Bone and Joint Surgery A*.

[3] Hüfner T, Kfuri M, Galanski M (2004). New indications for computer-assisted surgery: tumor resection in the pelvis. *Clinical Orthopaedics and Related Research*.

[4] Kojima T (2001). The usefulness and limits of magnetic resonance imaging in the differential diagnosis of pelvic tumors. *Oncology Reports*.

[5] Ozaki T, Flege S, Kevric M (2003). Osteosarcoma of the pelvis: experience of the Cooperative Osteosarcoma Study Group. *Journal of Clinical Oncology*.

[6] Fuchs B, Hoekzema N, Larson DR, Inwards CY, Sim FH (2009). Osteosarcoma of the pelvis: outcome analysis of surgical treatment. *Clinical Orthopaedics and Related Research*.

[7] Cartiaux O, Docquier PL, Paul L (2008). Surgical inaccuracy of tumor resection and reconstruction within the pelvis: an experimental study. *Acta Orthopaedica*.

[8] Stiehl JB, Heck DA (2008). Computer-assisted surgery: basic concepts. *Instructional course lectures*.

[9] Gautier E, Bächler R, Heini PF, Nolte LP (2001). Accuracy of computer-guided screw fixation of the sacroiliac joint. *Clinical Orthopaedics and Related Research*.

[10] Hüfner T, Pohlemann T, Tarte S (2002). Computer-assisted fracture reduction of pelvic ring fractures: an in vitro study. *Clinical Orthopaedics and Related Research*.

[11] Reijnders K, Coppes MH, van Hulzen ALJ, Gravendeel JP, van Ginkel RJ, Hoekstra HJ (2007). Image guided surgery: new technology for surgery of soft tissue and bone sarcomas. *European Journal of Surgical Oncology*.

[12] Wong KC, Kumta SM, Chiu KH (2007). Computer assisted pelvic tumor resection and reconstruction with a custom-made prosthesis using an innovative adaptation and its validation. *Computer Aided Surgery*.

[13] Enneking WF, Dunham WK (1978). Resection and reconstruction for primary neoplasms involving the innominate bone. *Journal of Bone and Joint Surgery A*.

[14] Paul L, Cartiaux O, Docquier PL, Banse X (2009). Ergonomic evaluation of 3D plane positioning using a mouse and a haptic device. *International Journal of Medical Robotics and Computer Assisted Surgery*.

[15] Zagars GK, Ballo MT, Pisters PWT, Pollock RE, Patel SR, Benjamin RS (2003). Surgical margins and reresection in the management of patients with soft tissue sarcoma using conservative surgery and radiation therapy. *Cancer*.

[16] Paul L, Docquier PL, Cartiaux O, Cornu O, Delloye C, Banse X (2010). Selection of massive bone allografts using shape-matching 3-dimensional registration. *Acta Orthopaedica*.

[17] Brown GA, Firoozbakhsh K, DeCoster TA, Reyna JR, Moneim M (2003). Rapid prototyping: the future of trauma surgery?. *Journal of Bone and Joint Surgery A*.

[18] Moroni A, Caja VL, Sabato C, Zinghi G (1995). Surgical treatment of both-column fractures by staged combined ilioinguinal and Kocher-Langenbeck approaches. *Injury*.

[19] Docquier PL, Paul L, Cartiaux O, Banse X (2009). Registration accuracy in computer-assisted pelvic surgery. *Computer Aided Surgery*.

[20] Wong KC, Kumta SM, Antonio GE, Tse LF (2008). Image fusion for computer-assisted bone tumor surgery. *Clinical Orthopaedics and Related Research*.

[21] Jones BC, Sundaram M, Kransdorf MJ (1993). Synovial sarcoma: MR imaging findings in 34 patients. *American Journal of Roentgenology*.

[22] Bonniaud G, Isambert A, Dhermain F (2006). Image registration for radiation therapy: practical aspects and quality control. *Cancer/Radiotherapie*.

[23] Nakamura M, Stöver T, Rodt T (2009). Neuronavigational guidance in craniofacial approaches for large (para)nasal tumors involving the anterior skull base and upper clival lesions. *European Journal of Surgical Oncology*.

[24] Nemec SF, Peloschek P, Schmook MT (2010). CT-MR image data fusion for computer-assisted navigated surgery of orbital tumors. *European Journal of Radiology*.

[25] Giesel FL, Mehndiratta A, Locklin J (2009). Image fusion using CT, MRI and PET for treatment planning, navigation and follow up in percutaneous RFA. *Experimental Oncology*.

[26] Willems PWA, van der Sprenkel JWB, Tulleken CAF, Viergever MA, Taphoorn MJB (2006). Neuronavigation and surgery of intracerebral tumours. *Journal of Neurology*.

[27] Paul L, Docquier PL, Cartiaux O, Cornu O, Delloye C, Banse X (2008). Inaccuracy in selection of massive bone allograft using template comparison method. *Cell and Tissue Banking*.

[28] Routt MLC, Swiontkowski MF (1990). Operative treatment of complex acetabular fractures. Combined anterior and posterior exposures during the same procedure. *Journal of Bone and Joint Surgery A*.

[29] Mayr E, Moctezuma de la Barrera JL, Eller G, Bach C, Nogler M (2006). The effect of fixation and location on the stability of the markers in navigated total hip arthroplasty. A cadaver study. *Journal of Bone and Joint Surgery B*.

[30] Siston RA, Giori NJ, Goodman SB, Delp SL (2007). Surgical navigation for total knee arthroplasty: a perspective. *Journal of Biomechanics*.

[31] Keppler P, Gebhard F, Grützner PA (2004). Computer aided high tibial open wedge osteotomy. *Injury*.

[32] Haider H, Barrera OA, Garvin KL (2007). Minimally invasive total knee arthroplasty surgery through navigated freehand bone cutting: winner of the 2005 “hap” PAUL AWARD. *Journal of Arthroplasty*.

[33] Grimer RJ (2005). Surgical options for children with osteosarcoma. *Lancet Oncology*.

